# The effect of dialectical behaviour therapy in autism spectrum patients with suicidality and/ or self-destructive behaviour (DIASS): study protocol for a multicentre randomised controlled trial

**DOI:** 10.1186/s12888-020-02531-1

**Published:** 2020-03-17

**Authors:** Anne Huntjens, L. M. C. Wies van den Bosch, Bram Sizoo, Ad Kerkhof, Marcus J. H. Huibers, Mark van der Gaag

**Affiliations:** 1 Parnassia Psychiatric Institute, The Hague, The Netherlands; 2 Department of Clinical Psychology, VU University, Amsterdam, The Netherlands; 3 Amsterdam Public Mental Health Research Institute, Amsterdam, The Netherlands; 4 Dialexis, Nijmegen; Synthis, Deventer, The Netherlands; 5 Centre for Developmental Disorders, Dimence Institute of Mental Health, Deventer, The Netherlands

**Keywords:** Autism, Suicidality, Self-destructive behaviour, Dialectical behaviour therapy

## Abstract

**Background:**

Many persons with autism spectrum disorder (ASD) are treated in long-term specialised care. In this population, suicidal behaviour troubles patients, families, and specialists in the field because it is difficult to treat. At present, there is no documented effective therapy for suicidal behaviour in ASD (Autism Research 7:507-521, 2014; Crisis 35:301-309, 2014). Dialectical Behaviour Therapy (DBT) is an efficacious treatment programme for chronically suicidal and/or self-harm behaviour in patients with Borderline Personality Disorder (J Psychiatry 166:1365-1374, 2014; Linehan MM. Cognitive behavioural therapy of borderline personality disorder. 1993). This study will evaluate the efficacy of DBT in persons with ASD and suicidal/ self- destructive behaviour in a multicentre randomised controlled clinical trial.

**Method:**

One hundred twenty-eight persons with autism and suicidal and/or self-harming behaviour will be recruited from specialised mental healthcare services and randomised into two conditions: 1) the DBT condition in which the participants have weekly individual cognitive behavioural therapy sessions and a 2.5 h skills training group session twice per week during 6 months, and 2) the treatment as usual condition which consists of weekly individual therapy sessions of 30–45 min with a psychotherapist or social worker. Assessments will take place at baseline, at post-treatment (6 months), and after a follow-up period of 12 months. The mediators will also be assessed at 3 months. The primary outcome is the level of suicidal ideation and behaviour. The secondary outcomes are anxiety and social performance, depression, core symptoms of ASD, quality of life, and cost-utility. Emotion regulation and therapeutic alliance are hypothesised to mediate the effects on the primary outcome.

**Discussion:**

The results from this study will provide an evaluation of the efficacy of DBT treatment in persons with ASD on suicidal and self-harming behaviour. The study is conducted in routine mental health services which enhances the generalisability of the study results to clinical practice.

**Trial registration:**

ISRCTN96632579. Registered 1 May 2019. Retrospectively registered.

## Background

Recent epidemiological data indicate that Autism Spectrum Disorder (ASD) occurs in 98 out of 10.000 individuals in the general population [1]. Suicidal ideation, self-harm, and suicide attempts are relatively prevalent in autism spectrum disorders with a prevalence from 11 to 66% for suicidal ideation, 1 to 35% for suicide attempts [2, 3]. Premature death by suicide is 7 times more often in persons with ASD [4]. ASD with depression has even higher rates with 85% suicidal ideation and 49% suicidal plans and attempts [5, 6]. Kato and colleagues (2013) stated that “individuals with ASD who have attempted suicide (a) had persistent rather than spontaneous stressors, (b) used more lethal means, and (c) were less connected to psychiatric services than the general population” [7]. This suggests that individuals with ASD may actually be more likely to succeed in their first suicide attempt [7].

Persons with ASD are known to have problems with emotion regulation [8, 9]. Disturbances of affective contact are a core feature of autism [9] and are strongly associated with suicidal ideation and attempts [10–12]. Cassidy and colleagues showed that depression is an important risk factor for suicidality and that social isolation is also associated with suicidal behaviour in autism [6]. Furthermore, ASD is characterised with deficits in expression of feelings and thoughts [10] and anxiety is the most prevalent comorbidity [10, 13]. Both are strongly associated with suicidality. There is no evidence-based treatment available to diminish suicidal ideation and behaviour for this population [10, 14].

In Borderline Personality Disorder, Dialectical Behavioural Therapy (DBT) is an empirically validated treatment for chronically suicidal and/or self-destructive behaviour in adolescent and adult patients [15, 16]. DBT focusses on enhancing emotion regulation skills and encompasses individual therapy, skills training in a group, therapist consultation, and occasional telephone consultation [17].

ASD and BPD share many characteristics: Problems with emotion dysregulation [18]; major problems in interpersonal relationships, identity disturbance, impulsivity, recurrent suicidal behaviour, gestures of threats, affective instability due to marked reactivity of mood, chronic feelings of emptiness, inappropriate intense anger or difficulty controlling anger, and transient, stress-related paranoid ideation and the inability to inhibit various urges, impulses, behaviours, or desires" [19]. DBT addresses these issues and teaches self-regulation, change skills, and skills for self-acceptance and the acceptance of others. The strict and repeated behavioural characteristics of DBT, as well as its focus on building emotion regulation skills, will be especially beneficial for the ASD population due to the autistic preference for predictable instructions and rules of conduct.

A documented mediator is the therapeutic alliance defined as the collaborative bond between therapists and patients [20]. The therapeutic relationship in DBT is considered as a dialectical therapeutic alliance in which the therapist can coach the patient in his/her skills to regulate his/her affect and behaviour [21]. This collaborative agreement between the therapist and the client about the goals and tasks of therapy will also support the preferences of ASD patients for predictable instruction and rules of conduct [22]. The development of a strong alliance between therapists and patients is thought to be especially important for effective intervention [23]. Another mediator is emotion regulation. Emotion dysregulation in ASD is associated with restricted and repetitive behaviour; core features of ASD [18]. Problems with emotion regulation can lead to suicidality [9, 12, 24]. The enhancement of emotion regulation in therapy, such as DBT, may mediate the effects of therapy on suicidality. DBT enhances emotion regulation as it teaches participants to observe and label events, thoughts, emotions, and bodily sensations in a non-judgmental, accepting way [21]. This may be helpful in coping with strong emotions and reducing depression, anxiety, and dysfunctional thoughts and acts [25, 26].

Alexithymia is a trait-like characteristic which causes difficulties in describing subjective emotional experiences. Recent research suggests that individuals with ASD also have high levels of alexithymia, with a prevalence of 40–65% reported in adults with ASD [27–29]. This may moderate therapy effects in a negative way.

In summary the central theme of this study is to determine the efficacy of a short term DBT treatment in persons with ASD and suicidal and/or self-harming behaviour and the mechanisms of change and contextual factors.

### Objectives

#### Primary objective

The primary objective of this study is to determine the efficacy of DBT treatment in persons with ASD on suicidal and/or self-harming behaviour.

- ► *Hypothesis 1*: DBT treatment is effective in decreasing suicidal and/or self-harming behaviour in the persons with ASD as measured by the combined score on Suicidal Ideation Attributes Scale (SIDAS) [30] and Lifetime Parasuicide Count (LPC) [31] in the first 6 months of treatment, compared to treatment as usual (TAU), and that this difference between the groups is sustained at 12 months.

#### Secondary objectives

The secondary objectives are to determine the efficacy of DBT versus TAU on anxiety, social performance, depression, and quality of life. Further, an economic evaluation will determine the cost-utility of DBT treatment.

- ► *Hypothesis 2*: DBT reduces social anxiety as measured by Social Interaction Anxiety Scale (SIAS) [32].
- ► *Hypothesis 3*: DBT enhances social functioning as measured by Personal and Social Performance Scale (PSP) [33, 34].
- ► *Hypothesis 4*: DBT reduces depression symptoms as measured by Beck Depression Inventory (BDI-II) [35, 36].
- ► *Hypothesis 5*: DBT enhances quality of life as measured by Manchester Short Assessment of Quality of Life (MANSA) [37] and by EuroQol Group (EQ- 5D) [38].
- ► *Hypothesis 6*: DBT changes severity of ASD symptoms as measured by Social Responsiveness Scale (SRS-A) [39].
- ► *Hypothesis 7*: DBT treatment improves healthcare costs and productivity losses as measured by Treatment Inventory of Cost is Patients with Psychiatric disorders (TIC-P short) [40].

### Other secondary objectives

In the current study we will conduct additional research to explore the mechanisms of change related to the treatment outcome variables. It is hypothesised that both emotion regulation and therapeutic alliance mediate the effects of therapy. The level of alexithymia is hypothesised to moderate the effects of therapy.

## Methods/design

### Design

This study is a single-blind multicentre randomised controlled trial with two arms: dialectical behavioural therapy (DBT) versus treatment as usual (TAU). TAU consists of weekly sessions of 30–45 min with a psychotherapist or social worker. The two groups will be compared at baseline, post-treatment (6 months), and at follow-up (12 months). The mediators will also be assessed mid-treatment at 3 months. All analyses will be performed according to the intention-to-treat principle. This trial meets all the requirements of the SPIRIT Protocol Checklist which provides guidance for protocols of clinical trials [41]. Checklist is provided in Additional file 1.

### Recruitment and study procedures

Adult participants (≥18) will be recruited from outpatient units in Dutch mental healthcare centres with specialised departments in the treatment and care for autism spectrum disorder: Parnassia Psychiatric Institute (Den Haag and Rotterdam), Rivierduinen Psychiatric Institute (Leiden), and Lentis Psychiatric Institute (Groningen). All participants had been diagnosed with ASD by experienced clinicians according to national guidelines, prior to inclusion [42]. Written informed consent will be obtained from each participant.

**Fig. 1:**
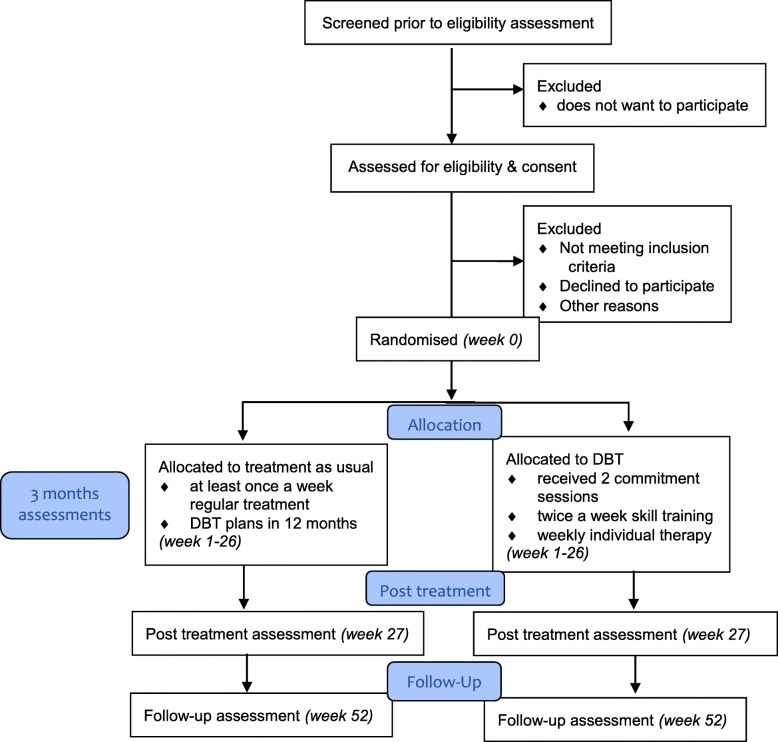
Trial flow *diagram*. Assessments will take place at baseline, at post-treatment (6 months), and after a follow-up period of 12 months. The mediators will also be assessed at 3 months

Patients in participating mental health centres with a diagnosis of ASD and suicidality will be informed about the study by their treating specialist (see Fig. 1) and will be asked for permission to be contacted by the researchers. Potential participants will be phoned by a research assistant and provided with further information about the study.

Patients will have 2 weeks to decide about their participation. If the patient is willing to participate, informed consent will be signed. To verify the clinical diagnosis of ASD, we will use a cut-off ≥70 on the 28-item Short version of the AQ-28 [43, 44]. Patients who are willing to participate will be screened for suicidal and/or self-destructive behaviour and level of suicidality/self-harming behaviour in the last year. After screening, baseline data will be collected and participants will be randomly allocated to one of the two study groups. Participants who are randomised to DBT will start with two commitment sessions. After commitment is reached, the goals and targets of treatment will be discussed. A personalised treatment contract will be formulated and signed by the individual therapist and the patient. After 6 months, post-treatment assessment will be performed in both conditions. After 12 months, a follow-up measurement for all primary and secondary outcomes will be performed in both conditions. The mediators will also be assessed mid-treatment at 3 months.

### Inclusion criteria

1. Age between 18 and 65 years
2. Meets DSM-5 criteria for autism spectrum disorder [45]
3. Suicidal ideation (SIDAS score ≥ 21) [30] and/or level of suicidality/self-harming behaviour rated as severe on the LPC (score = 2 on any item) [31]
4. Outpatient status

### Exclusion criteria

1. IQ < 80 (only assessed with WAIS-IV if baseline testing is difficult because of intellectual deficits)
2. Addiction to illicit drugs and in need of clinical detoxification
3. Insufficient mastery of the Dutch language

### Randomisation

One block of 16 random assignments will be generated for each participating mental health centre. Each block will have 8 assignments for each condition: DBT or control group, treatment as usual. If a centre includes more patients, new blocks of random assignments will be generated. The blocks and random assignments will be generated using a scientific randomisation programme (www.randomizer.org) by the independent randomisation bureau of Parnassia Psychiatric Institute. Participants will be informed about their allocation by mail. The therapists will be informed about the participation of their client and about which condition the client has been allocated.

### Study interventions

The therapy consists of DBT skills training [46], individual cognitive behavioural therapy [47], and weekly meetings with the consultation team for all trainers and therapists for 1 h. The complete staff will be supervised once a month by a fully certified trainer and supervisor (WvdB).

Two adaptations were made to the standard DBT program for this study protocol. First, the telephone consultation (if needed) was limited to the therapist’s availability, which means that not all patients could use 24-h consultation. Second, the treatment period is limited to half of the original treatment time (1 year) because of the expected reduced concentration span. The preference for repetitive instructions in people with ASD, in combination with the concentration span, led to the decision to offer the skills training twice a week [48].

Individual cognitive behavioural therapy will take place on a weekly basis. The order and topics of each session are pre-determined based on Linehan’s hierarchy [15, 16]: suicidal and self-destructive behaviour, therapy interfering behaviour, quality of life interfering behaviour, and generalisation of the skills taught in the training. Each therapy session starts with self-report diary cards [49] that describe problematic behaviours and behaviours that influence the primary goals such as alcohol and drug use, the urge to self-harm, substance abuse, dissociation, level of applied skills, etc. Two skills training groups will take place each week. The skills taught are self-regulation skills, change skills, self-acceptance and acceptance of others, mindfulness, interpersonal effectiveness, and skills to cope with crises. When a meeting is missed, the participant will be asked to catch up by watching video recordings that are made of all the trainings sessions. The therapists keep track of self-reported watching by the experimental subjects. We consider that being in the programme for 5 months is the minimum number of sessions required for an adequate amount of intervention. All therapists are either psychologists or psychiatrists and skill trainers are psychologists, registered nurses, or social workers (*n* = 28). They will have 5 days of training in DBT by a licensed DBT therapist, teacher, supervisor, and trainer (AH).

The control group will receive treatment as usual. This can be any form of treatment for suicidality in autism which is common within mental health care and is delivered in weekly sessions of 30–45 min with a psychotherapist or social worker. Therapists will only participate in one of the conditions.

### Outcomes and measures

An overview of all outcome measures and the different assessment moments in which they will be used is shown in Table 1.

**Table 1:** Schedule of Enrolment, Interventions, and Assessment

		Study Period			
	Enrolment	Allocation		Post-allocation	
TIMEPOINT	Screening	Baseline	3 months	Post-treatment	Follow- up
**ENROLMENT**					
**Eligibility screen**	x				
**Informed consent**	x				
**Allocation**		x			
**INTERVENTIONS:**					
***SIDAS***	x	x		x	x
***LPC***	x	x		x	x
***AQ-Short***	x				
***BDI-II***		x		x	x
***Demographics***		x			
***DERS***		x	x	x	x
***EQ-5D***		x		x	x
***MANSA***		x		x	x
***PSP***		x		x	x
***SIAS***		x		x	x
***SRS-A***		x		x	x
***TAS-20***		x		x	x
***TIC-P short***		x		x	x
***WAV-12***^***a***^		x	x	x	x

*SIDAS* Suicidal Ideation Attributes Scale, *LPC* Life time Parasuicide Count, *AQ Short* Autism spectrum quotient (short), *BDI-II* Beck Depression Inventory – Second Edition, *DERS* Difficulties in Emotion Regulation, *EQ-5D* EuroQol 5 dimensions, *MANSA* Manchester short Assessment of quality of life, *PSP* Personal and Social Performance Scale, *SIAS* Social Interaction Anxiety Scale, *SRS-A* The Social Responsiveness Scale, *TAS* Toronto Alexithymia Scale, *TiC-P short* Trimbos/iMTA questionnaire for Costs associated with Psychiatric illness, *WAV-12* Working Alliance Inventory

^a^will be rated by participants and individual therapists after each completed session of the skills training module

#### Primary outcomes

The primary outcome will be assessed as the total score of the Suicidal Ideation Attributes Scale (SIDAS) plus the Lifetime Parasuicide Count (LPC).

##### Suicidal ideation attributes scale (SIDAS)

The Suicidal Ideation Attributes Scale is a measure of the severity of suicidal ideation. It consists of five items, each targeting an attribute of suicidal thoughts: frequency, controllability, closeness to attempt, level of distress associated with the thoughts, and impact on daily functioning. Scores ≥21 indicate high risk of suicide behaviour. The SIDAS demonstrated high internal consistency and good convergent validity [30].

##### Lifetime Parasuicide count (LPC)

The Lifetime Parasuicide Count is a structured, face to face interview for assessing information regarding lethality, intention to die, level of medical treatment received, and specific details of the participant’s first, most recent, and most severe episodes of parasuicide [31]. The LPC measures the level of self-injurious behaviours and suicide attempts. The scores of the SIDAS and LPC will be combined in order to evaluate the multiple aspects of suicidal mood and behaviour.

#### Secondary outcomes

##### Autism Spectrum quotient (AQ-short)

To verify the clinical diagnosis of ASD, we will use the 28-item short version of the Autism Spectrum Quotient (cut-off ≥70) [43, 44]. AQ-Short shows the total AQ-Short score and its two higher-order factors showed acceptable to good internal consistency. The AQ-Short is a reliable instrument for a quick assessment of quantitative autistic traits.

##### Social responsiveness scale (SRS-A)

The Social Responsiveness Scale is a 65-Item self-report questionnaire which assesses the presence and extent of autistic symptoms in adults population. The SRS-A has demonstrated high internal consistency (α = 0.95 and 0.94 in typical males and females, respectively) and test–re-test reliability (r = 0.8). The questionnaire was selected based on its excellent psychometric properties and its focus on deficits aligning with the social domain of ASD [39].

##### Social interaction anxiety scale (SIAS)

Interaction anxiety symptoms, such as fear of interaction with others, are assessed with the Social Interaction Anxiety Scale. The SIAS consists of 19 items that assess the tendency of fear and the avoidance of social situations. The SIAS has good psychometric properties [32].

##### Beck depression inventory (BDI-II)

Severity of depression symptoms will be assessed with the Beck Depression Inventory second edition. The BDI-II has good psychometric properties and was found to be a reliable and valid measure [35, 36].

##### Manchester short assessment of quality of life (MANSA)

Quality of life will be assessed by the Manchester Short Assessment of Quality of Life. The MANSA particularly assesses satisfaction with life as a whole and with several life domains. The psychometric properties of the MANSA, both concurrent validity and reliability, appear satisfactory [37].

##### Personal and social performance scale (PSP)

The Personal and Social Performance Scale assesses social functioning. The PSP scale is a rating scale that measures personal and social functioning in the domains of socially useful activities (for example, work and study), personal and social relationships, self-care, and disturbing and aggressive behaviours. The PSP showed good (> 0.80) interrater reliability and test–retest reliability [33, 34].

##### Difficulties in emotion regulation scale (DERS)

The Difficulties in Emotion Regulation Scale is a brief, 36-item, self-report questionnaire designed to assess multiple aspects of emotion dysregulation [50, 51].. The DERS has also exhibited good construct validity in adult psychiatric patients. Specifically, the measure has demonstrated sensitivity to change due to successful clinical intervention [52, 53].

##### Working Alliance questionnaire (WAV-12)

The Working Alliance Questionnaire is a validated Dutch 12-item revised version of the Working Alliance Inventory (WAI). This is a self-report instrument to assess the quality of the working relationship as perceived by the client and the therapist [54]. The WAV-12 has demonstrated good internal consistency reliability (α = 0.82–0.85) and good construct validity (Goodness-of-Fit index 0.90) [55].

##### Toronto alexithymia scale (TAS-20)

The Toronto Alexithymia Scale is a 20-item instrument that is one of the most commonly used measures of alexithymia. Demonstrates good internal consistency (Cronbach’s alpha = .81) and test-retest reliability (.77, *p* < .01). Validity: Research using the TAS-20 demonstrates adequate levels of convergent and concurrent validity [56].

##### EuroQol group (EQ- 5D)

The EQ-5D is a five-item measure of health status and quality of life with a score range from − 0.594 (worst possible health) to 1.0 (perfect health). The 243 possible health states on the EQ. 5D are evaluated against a normal population [38].

##### Treatment inventory of cost in patients with psychiatric disorders (TIC-P short)

The Trimbos/iMTA (Institute for Medical Technology Assessment) questionnaire for Treatment Inventory of Costs in Patients with psychiatric disorders short version is a self-report questionnaire designed for patients with a mental disorder. The questionnaire focuses on establishing direct medical costs and productivity costs during paid or unpaid work. The psychometric properties are reported to be adequate [40].

##### Demographics

Assesses basic personal, social, medical data, age, date of birth, country of birth, highest level of achievement in education, daily housing/living situation, DSM-5 diagnoses, substance abuse or dependence, current medication, years of illness of suicidality and autism.

##### The Wechsler adult intelligence scale – fourth edition; WAIS-IV

*Dutch version 2014:* is a comprehensive clinical instrument for assessing the intellectual abilities of older adolescents and adults [57].

### Sample size

To find an expected medium effect size of 0.50 with an alpha of 0.05 and power of 0.80, our studies requires 128 participants with 64 in each arm. The three participating mental health centres have four sites with mental health services and care for more than 3000 ASD patients. Estimates of suicide ideation and attempts in the population with ASD range from 20.4 to 10%, respectively [2]. If the prevalence of suicidal behaviour is 15%, then the participating centres have 450 eligible patients. A 30% recruitment rate will be enough for the needed 128 participants.

### Data analysis

The result of randomization will be checked by comparing baseline sociodemographic and clinical parameters between DBT and TAU condition, using univariate analyses. The study is intention-to-treat. So, all patients that have been randomized, will be in the analysis. If patients miss measurements, this will be dealt with LMM that uses expectancy maximisation to impute missing data. If patients miss four sessions in a row, the patient is considered a treatment dropout and therapists will stop pursuing the patients to continue treatment. Treatment drop-out will still be contacted for measurements, though. Treatment dropouts will still be part of the analysis in the intention-to-treat analysis. Study dropouts that have missing measurements will have the LMM imputation strategy to estimate scores and impute these bases on expectancy maximisation. If *p*-values are marginally, we will also use LOCF, last observations carried forward, as an imputation strategy to do a conservative sensitivity analysis. The primary outcome on the suicidality measures will be analysed using Linear Mixed Models (LMM) where condition is the fixed effect, measurement moment and site is a fixed factor, individual is a random effect with the baseline value as a covariate. The group x moment interaction will be assessed for post-treatment and follow-up results. We are aware that the SIDAS is used both in selection of patients and as an outcome measure and that regression to the mean may negatively impact on the interaction effect. The SIDAS score will probably not regress to normal score levels and cause a bottom effect. To cope with the regression to the mean, we compute the main effects of condition and time and the interaction of condition x time with baseline as a covariate. To identify mechanisms of change and the strength of the factors involved, both multilevel models and structural equation models will be used for mediation analyses. Mediation analysis with a multiple mediator model (multiple mediators in the same model) will be calculated with 5000 bootstraps using the PROCESS macro published by Hayes and Rockwood (2017) [58]. Moderators will be analysed using mixed modelling, in addition to the effectiveness analysis. Cost-utility analysis will also be performed.

### Data management

All data will be pseudomised during the study period. A hard copy of the data will be stored anonymously at the Secured Data Centre of the Department of Scientific Research and Innovation of Parnassia Psychiatric Institute and will only be available to the researchers involved in the study. A data monitor from Parnassia Psychiatric Institute is appointed for the study. Only the monitors and the principal researchers will have access to the final dataset. Data will be stored after the trial for 15 years. A Data Monitoring Committee is not needed since this study is considered low risk.

### Ethical and safety considerations

A number of patients with autism co-wrote the workbook on dialectical behaviour therapy with the first author. The trial is conducted with patients and therapists from routine mental health services. The burden of the intervention will be assessed and evaluated by participants. A summary of the main findings at the end of the study will be sent to all participants. Participation is completely voluntary, and participants can withdraw from participation at any time for any reason. Participants who decide to stop therapy will be encouraged to continue to participate in the assessments. There is no relevant risk for participating DBT. The use of dialectical behavioural therapy has not led to risks. DBT protocol uses suicide risk assessment. DBT therapists in the study have been trained in the assessment of suicide risk. This includes assessment questions for gathering information to determine the level of risk and action steps that can be taken to ensure safety. The assessment is also evaluating risk factors, warning signs and psycho-social stress factors of self-destructive, and suicidal behaviour. Patients in DBT use diary cards, which track all relevant behaviours during the past week. On the diary card, the patient gives a score daily, from 0 to 5, on the domains ‘severity of suicidal ideation’ and ‘urge for self-injury’. On the frequency list, the patient reports daily the times he performs self-injury practices. Potential serious adverse events (SAE) will be reported to the Medical Ethics Committee of the VU University Medical Centre.

### Fidelity

All sessions will be videotaped. The treatment integrity in DBT will be assessed by video recordings of each session. Videos will then be systematically evaluated by trained adherence coders. The DBT therapists will have four-hour group supervisions each month. Supervision is provided by a researcher (WvdB) who is the head of the Dutch DBT Association and has received extensive training from Dr. Linehan.

### Unblinding

The study is single-blinded, meaning that research assistants who facilitate the outcome assessments will be kept blinded regarding randomisation and allocation of the participants. Participants will be regularly instructed not to tell the research assistants which group they are allocated to. If a research assistant is accidentally unblinded during a measurement, that measurement will be stopped. A new appointment will then be made with another blinded research assistant**.**

### Adverse events

The rules and regulations of the Medical Ethics Committee of the VU University Medical Centre concerning adverse events will be followed. All participants will be insured in case any harm is caused related to trial participation.

### Protocol modifications

Any modifications of the protocol will be formally amended and submitted to the Medical Ethics Committee of the VU University Medical Centre. The rules of the METC for communicating this change to relevant parties will be followed.

### Dissemination

Trial results will be published in peer-reviewed international journals and will be presented at national and international conferences. If DBT is found to efficacy reduce suicidality and self-destructive behaviour in autism patients, the treatment manual for this target group will be made available.

## Discussion

Because there are no empirical data to guide practitioners in the treatment of suicidality in persons with ASD, it is important to determine if DBT is an effective intervention to reduce or prevent suicide ideation and attempts [2, 5, 6]. Given that DBT has been found to effectively decrease parasuicidal behaviour and other areas of behavioural dyscontrol in the treatment of emotion regulation problems in patients with a diversity of disorders [59], we hypothesise that DBT will also be effective in decreasing suicidality for individuals with autism.

This study has several strengths. Foremost, it is the first single-blind randomised controlled clinical trial to examine the efficacy of DBT in people with autism and suicidality. Furthermore, this study is conducted in routine mental health services which enhances the generalisability of the study results to regular clinical practice. Finally, our sample is relatively large and consists of both men and women.

No major difficulties are expected with regard to the recruitment of persons with autism. However, due to the traditional cautiousness of some therapists, recruitment may be hindered as therapists may be more reluctant to recommend this treatment to their clients. Therefore, information sessions for therapist about the study will be held at the participating mental health centers to help facilitate recruitment. Since suicidality is known to be an enduring problem in people with ASD, a main limitation of this study is the short follow-up. It could also be argued that the contact time in the control condition (TAU) will not match that of DBT. However, TAU consists of standard care for persons with ASD and suicidality and the aim of the study is to investigate the efficacy of DBT treatment for persons with ASD and suicidality or self -destructive behaviour. The comparison of DBT treatment and a control condition will provide further information about the efficacy of DBT for this population. If DBT is found to effectively reduce suicidality and self-destructive behaviour in autism patients, new guidelines for the conceptualisation and the use of DBT can be drafted.

Another limitation is the recruitment and suitability of therapist who are willing to work with this group and with DBT. In addition to this, the qualifications of the TAU therapists are not clear. Regardless of the fact that a number of persons with autism assisted in writing the DBT workbook, the fact that the treatment period is limited to half of the original treatment (1 year) may also influence the treatment.

Suicidality in people with autism remains poorly understood and vastly under-researched. Studies have emphasized the need for more research on the efficacy of prevention strategies and treatment interventions. This protocol represents a proof of concept study that is intended to evaluate the efficacy of DBT treatment in patients with ASD and suicidal behaviour in routine mental health care settings. If the program is found to be effective for the patients and for the clinics, then it may be implemented in other clinics as well.

### Trial status

This study is registered and approved by the Medical Ethics Committee of the VU University Medical Centre (METC number: 2017.547/ NL59497.029.17) on March 2018.

The recruitment period for the trial started on August 2018 and is predicted to continue until July 2020.

On 2019-May-20, amendment on the protocol version 2, took place with the reason *addition of participating centre*.

International trial-registered took retrospectively place on 1 May 2019 at the International Standard Registered Clinical/Study Number (ISRCTN) under the title “Reducing suicidality in autism-spectrum patients using dialectical behaviour therapy”, identification code ISRCTN96632579.

The study is ongoing and in the data collection stage.

## Supplementary information

**Additional file 1.** Checklist Standard Protocol Items: Recommendations for Intervention Trials (SPIRIT).

## Data Availability

The datasets used and/or analysed during the current study are available from the corresponding author on reasonable request.

## Abbreviations

AQ Short: Autism spectrum quotient (short)
BDI-II: Beck Depression Inventory – Second Edition
BPD: Borderline Personality Disorder
DBT: Dialectical Behavioural Therapy
Demographics: Demographic characteristics
DERS: The Difficulties in Emotion Regulation
DSM-5: Diagnostic and Statistical Manual of Mental Disorders, fifth edition
EQ-5D: EuroQol 5 dimensions;
IC: Informed consent
LPC: Lifetime Parasuicide Count
MANSA: Manchester Short Assessment of quality of life
PSP: Social functioning
RCT: Randomised controlled trial
SIAS: Social Interaction Anxiety Scale
SIDAS: Suicidal Ideation Attributes Scale
SPIRIT: Standard Protocol Items; recommendation for Interventional Trials
SRS-A: The Social Responsiveness Scale
TAS: Toronto Alexithymia Scale
TAU: Treatment as usual
TiC-P short: Trimbos/iMTA questionnaire for Costs associated with Psychiatric illness
WAV-12: Working Alliance Inventory
